# Primary Neonatal Diaphragmatic Abscess

**Published:** 2015-01-10

**Authors:** Mohamed Zouari, Mohamed Jallouli, Afef Ben Thabet, Mahdi Ben Dhaou, Abdellatif Gargouri, Riadh Mhiri

**Affiliations:** 1 Department of Pediatric Surgery, HediChaker Hospital, 3029 Sfax, Tunisia.; 2 Department of Neonatology, HediChaker Hospital, 3029 Sfax, Tunisia.

**Keywords:** Abscess, Diaphragm, Liver, Neonate

## Abstract

Neonatal diaphragmatic abscesses are extremely rare and they usually develop by direct extension from a liver abscess. The first case of primary diaphragmatic abscess in a neonate is reported and the difficulties of diagnosing this rare entity are discussed.

## INTRODUCTION

Intra-abdominal abscesses are rare in neonates, and they could occur spontaneously or as a complication of abdominal pathologies and surgical procedures [1]. Neonatal diaphragmatic abscesses are extremely rare and they usually develop by direct extension from a liver abscess [1-4]. We report a case of primary diaphragmatic abscess in a neonate. To the best of our knowledge, such localization has not been previously reported for neonatal intra-abdominal abscesses.

## CASE REPORT

We describe a male newborn at 29 weeks gestation with a birth weight of 1100 g and delivered by primary cesarean section due to eclampsia. He was admitted to the neonatal intensive care unit for neonatal respiratory distress syndrome. Umbilical venous catheter and Broviac catheter were placed for three days and twelve days, respectively. The evolution was initially favorable.

On day 32, the baby also developed fever in addition to respiratory distress suspecting a nosocomial infection. He required intubation and initiation of mechanical ventilation. Blood cultures were drawn, and ampicillin and cefotaxime were started. Laboratory investigations revealed a white cell count of 11.2 × 109/l, neutrophils 9.3 × 109/l, platelets 126 × 109 /l, C-reactive protein 110 mg/l and hemoglobin of 11.9 g/dl. Blood cultures, analysis of the cerebrospinal fluid, and urinalysis were negative. The chest X-ray showed a right basilar opacity. Ultrasound scan showed 2 collections in the liver suggesting a hematoma. Thoraco-abdominal computed tomography scan showed a 5.5 X 4.2 X 3.2 cm abscess in the liver dome with trans-diaphragmatic spread (Fig.1A, 1B) but the origin of the abscess could not be identified (pulmonary or hepatic). We retained the diagnosis of liver abscess and a right subcostal laparotomy was performed. No abscess in the liver was found. The bare area of the liver was opened and exposed but there was no liver lesion. Then, a right posterolateral thoracotomy was done; the right lung had normal appearance without pleural effusion. We found a 5x5 cm fluctuant swelling in the right diaphragmatic dome. Six ml of yellowish pus was drained by puncture-aspiration. Surgical drainage was performed; a drain was left in abscess cavity for five days. Culture of pus was positive for staphylococcus aureus. The baby remained hospitalized for 3 weeks postoperatively because of a diaphragmatic hypokinesis. Follow-up ultrasound before discharge showed resolution of the diaphragmatic abscess.

**Figure:**
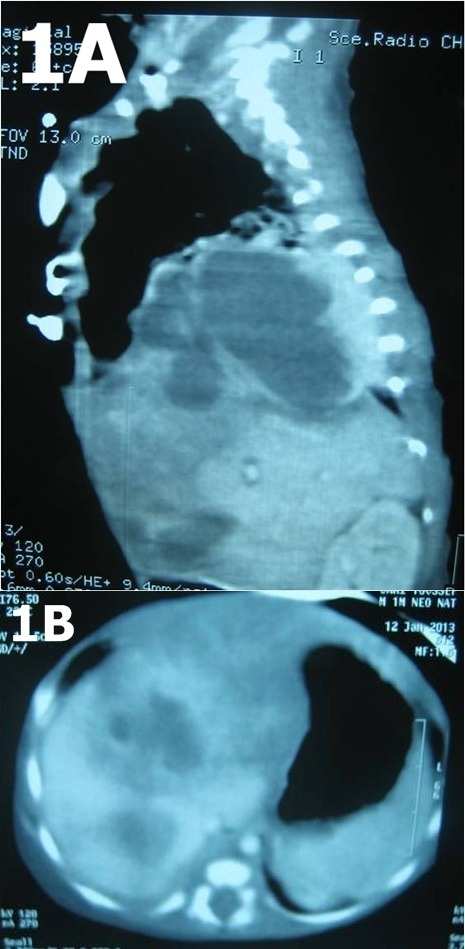
Fig. 1A, 1B: Thoraco-abdominal computed tomography scan showed a large abscess in the liver dome.

## DISCUSSION

Intra-abdominal abscesses are uncommon in neonates. The literature review shows that liver abscesses were the most common location for intra-abdominal abscesses. Risk factors include blood sepsis, umbilical catheterization, central parenteral nutrition catheters, necrotizing enterocolitis, surgery, and prematurity [1-4]. Neonatal diaphragmatic abscesses are extremely rare and they usually develop by direct extension from a liver abscess [1-3]. We reported the first case of a primary diaphragmatic abscess in a neonate.

The diaphragm harbors a rich vascular network including arteries forming a branch-like pattern on its superior and inferior surfaces, veins and lymphatic plexuses [5]. Therefore, we suggest that the diaphragmatic abscess was caused from hematogeneous spread via the diaphragmatic arteries. It can also be argued that it could have been a congenital lesion of the diaphragm that acted as a nidus for the infection to lodge and fester. Such lesions are extremely rare in children and only few cases have been reported in the literature [6, 7].

In our case, the diaphragmatic location of the abscess was not detected on CT scan. Diaphragmatic lesions are very difficult to detect on CT scan and, unless a clear splitting of the diaphragm is identified, the correct location of the lesion can be recognized only at surgical exploration [7]. We believe that magnetic resonance imaging (MRI) could have possibly better defined the exact site of this exceptional localization of thoraco-abdominal abscesses.

## Footnotes

**Source of Support:** Nil

**Conflict of Interest:** None

